# A New Powerful Method for Site-Specific Transgene Stabilization Based on Chromosomal Double-Strand Break Repair

**DOI:** 10.1371/journal.pone.0026422

**Published:** 2011-10-17

**Authors:** Artem Tkachuk, Maria Kim, Oksana Kravchuk, Mikhail Savitsky

**Affiliations:** 1 Group of Telomere Biology, Institute of Gene Biology, Russian Academy of Sciences, Moscow, Russia; 2 Center for Medical Studies of Oslo University, Moscow, Russia; Oregon State University, United States of America

## Abstract

Transgenic insects are a promising tool in sterile insect techniques and population replacement strategies. Such transgenic insects can be created using nonautonomous transposons, which cannot be transferred without a transposase source. In biocontrol procedures where large numbers of insects are released, there is increased risk of transgene remobilization caused by external transposase sources that can alter the characteristics of the transgenic organisms lead horizontal transgene transfer to other species. Here we describe a novel, effective method for transgene stabilization based on the introduction of directed double-strand breaks (DSB) into a genome-integrated sequence and their subsequent repair by the single-strand annealing (SSA) pathway. Due to the construct's organization, the repair pathway is predictable, such that all transposon and marker sequences can be deleted, while preserving integration of exogenous DNA in the genome. The exceptional conservation of DNA repair pathways makes this method suitable for a broad range of organisms.

## Introduction

Transgenic technologies have facilitated the generation of genetically modified insects that can be used to improve conventional biocontrol methods to manage agricultural pests and disease vectors [1], [2], [3], [4], [5], [6]. Germline transformation with transposon-based vectors remains the most suitable gene-delivery system for producing transgenic insects. The most common in current use are vectors based on *P-* element *piggyBac*, *mariner*, *Minos*, *hobo* and *Herme*s [4], [7]. Such standard nonautonomous vectors contain target DNA surrounded by transposon sequences that include inverted terminal repeats (ITRs), which are necessary for transposase binding and effective cutting and pasting processes. Stable integration is crucial for the maintenance and consistent expression of the transgene, therefore a transiently provided transposase-coding gene is removed from the transgenic strain. In small scale laboratory studies using genetically pure lines, accidental transgene remobilization is unlikely, but this risk increases during mass rearing and after insect release. The same or related transposase can be introduced into modified insects from the wild population, leading to transgene relocation or loss [8], [9]. The major ecological concern is the possibility of horizontal transfer of the transgene to other species [8].

To render transposons immobile, deletion of just one of the ITRs would be sufficient. Existing methods of postintegration transgene stabilization are based on vectors that carry an additional ITR [10], [11]. Such complex vectors are made up of two vectors, with the smaller of the two contained within the larger, and the one of the ITRs shared by both. After a full-size construct is inserted in the genome, an additional round of transposase-mediated remobilization is required, during which the smaller of the two vectors is more likely to be cut. As a result, the stabilized transgene with a single ITR remains in the genome. The use of two additional ITRs allows for complete deletion of transposon sequences [12], [13]. The effectiveness of the full-size construct's integration is not high due to the preference of the transposase for smaller vectors, but the introduction of an additional ITR by site-specific integration in FRT- or attP-containing platforms can circumvent this problem [14], [15]. However, the necessity of transposase-mediated remobilization renders the method susceptible to the specifics of transposon behavior, and, in particular, to transposase activity that must be sufficient for remobilization. On the other hand, transposase high activity may cause repeated transposon remobilization, resulting in uncontrolled mutations. Such mutations may negatively affect the survival of transgenic lines, which is an important parameter in population substitution programs.

We have designed a method that allows the complete removal of transposon sequences without an additional round of remobilization. The method has two stages: (i) site-specific vector insertion into a pre-integrated transgene (landing platform) by *phiC31*-mediated recombination; and (ii) introduction of DSB into the integrated sequence using I-SceI and I-CreI homing nucleases [16], [17]. The repair of DSB introduced between two direct repeats is usually carried out by the SSA pathway. In this case, only one repeat remains, and the sequence between them is deleted [18]. Therefore, if the transgene contains duplications of sequences flanking the landing platform, introduction of breaks between the direct repeats will lead to deletion of DNA sequences contained between them, including transposon termini.

## Results

**Figure 1:**
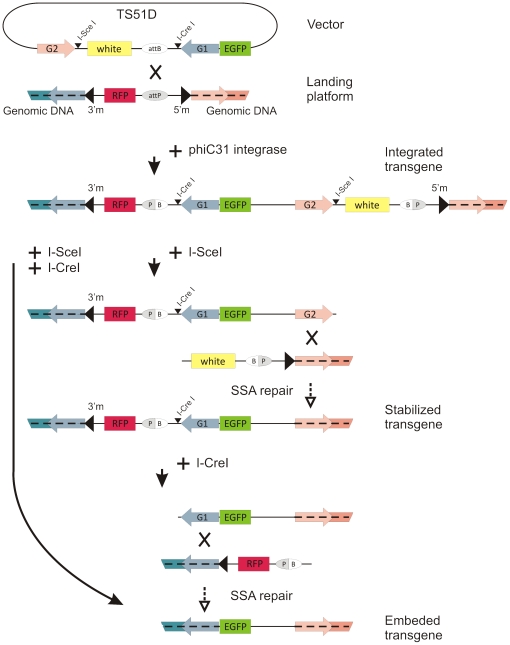
TS51D vector stabilization strategy after site specific integration in genome. Complete stabilization may be achieved in two steps (DSB introduction at I-SceI and then at I-CreI), or in one (simultaneous expression of two endonucleases). The breaks are repaired due to duplications of sequences flanking landing platform. Genomic sequences homologous to G1, G2 are highlighted corresponding to G1 (blue) and G2 (pink). Final insertion does not contain transposone sequences and, therefore, is immobile.

Insertion *M{3xP3*-*RFPattP}ZH-51D* [19], integrated in the genome of *Drosophila melanogaster* (2R: 10941803), was chosen as the landing platform. This *mariner*-based vector contains red fluorescent protein (RFP) driven by an artificial 3xP3 promoter, which induces strong RFP expression in the eye, and an attP site, which serves as the docking site for integration of *attB*-containing plasmids [19]. We constructed the *attB*-containing TS51D vector (**Fig. 1**) with the enhanced green fluorescent protein (EGFP) gene under control of the 3xP3 promoter selected as the sequence to be stabilized [20], [21]. *EGFP* was surrounded by *D. melanogaster* genomic sequences 998 (G1) and 645 bp (G2) in length, which flank the landing platform. Recognition sites for I-SceI and I-CreI homing nucleases were placed to introduce DSB into the transgene sequence. The TS51D vector also contains the marker gene *white*, which is responsible for red eyes in *Drosophila*.

The TS51D vector was integrated into the landing site by *phiC31-*mediated germline transformation [19], [22], [23]. The locus was as follows: genomic G1-homologous sequence – *mariner* 3′ITR – RFP – I-CreI site and G1 – EGFP – G2 and I-SceI site – *white* – *mariner* 5′ITR – genomic G2-homologous sequence (**Fig. 1**). The orientation of G1 and G2 is crucial, as is the position of I-SceI and I-CreI, so it is critical that G1 and G2 after integration of TS51D are collinear to the homologous sequences in the genome, and that I-SceI and I-CreI sites are located between the duplicated sequences.

Flies containing TS51D were crossed to flies carrying heat-inducible *I-SceI*, which produced F2 generation where 37.55% and 5.93% of flies lost the *white* marker following strong and light heat shock, respectively (phenotypic class R+G+W-) (**Table 1**). In this case, DSB occurred between G2 and the *white* gene, with both the *white* and 5′ITR of *mariner* deleted by SSA repair (**Fig. 1**).

**Table 1:** Stability of TS51D, TS51D2xSce and TS58A2xSce transgene vectors at DSBs induction under different heat shock delivery as indicated by absence or presence of marker phenotypes.

	TS51D				TS51D2xSce			TS58A2xSce
	I-SceI		I-CreI^c^	I-SceI + I-CreI	I-SceI			I-SceI
phenotype	*strong*	*light*	*light*	*light*	*strong*	*intermediate*	*light*	*strong*
**W+(G+)R+** a	2332 (62,45%)	1015 (94.07%)	-	1703 (73.98%)	685 (58.65%)	1065 (87.52%)	1327 (95.6%)	1750 (45.88%)
**W-(G+)R+** a	1402 (37.55%)	64 (5.93%)	3135 (85.9%)	24 (1.04%)	146 (12.5%)	57 (4.68%)	8 (0.58%)	1086 (28.47%)
**W+G+R-**	-	-	-	571 (24.8%)	267 (22.86%)	76 (6.24%)	53 (3.82%)	675 (17.7%)
**W-G+R-**	-	-	513 (14.1%)	**4 (0.17%)**	**70 (5,99%)**	**19 (1.56%)**	0	**303 (7.94%)**
**stabilization** b	1402 (37.55%)	64 (5.93%)	3648 (100%)^c^	599 (26.02%)	483 (41.35%)	152 (12.49%)	61 (4.4%)	2064 (54.12%)
**total**	3734	1079	3648	2302	1168	1217	1388	3814

All vectors contain two recognition sites for homing endonucleases. DSBs can be induced in TS51D sequentially (columns I-SceI and I-CreI) or simultaneously (I-SceI + I-CeI). Two breaks are induced in TS51D2xSce and TS58A2xSce. For optimal induction of I-SceI strong heatshock is necessary, and for I-CreI – light one. Figures for full one-step transgene stabilization are in bold. Statistical analysis is provided in Supplementary Table S1.

a (G+) GFP expression in these phenotypes is masked by RFP expression.

b summary stabilization after deletion of at least one ITR (sum of **W-(G+)R+, W+G+R- and W-G+R-)**.

c DSB induction by I-CreI endonuclease was carried out in flies with already stabilized transgene (without 3′ITR and *white*).

**Figure 2:**
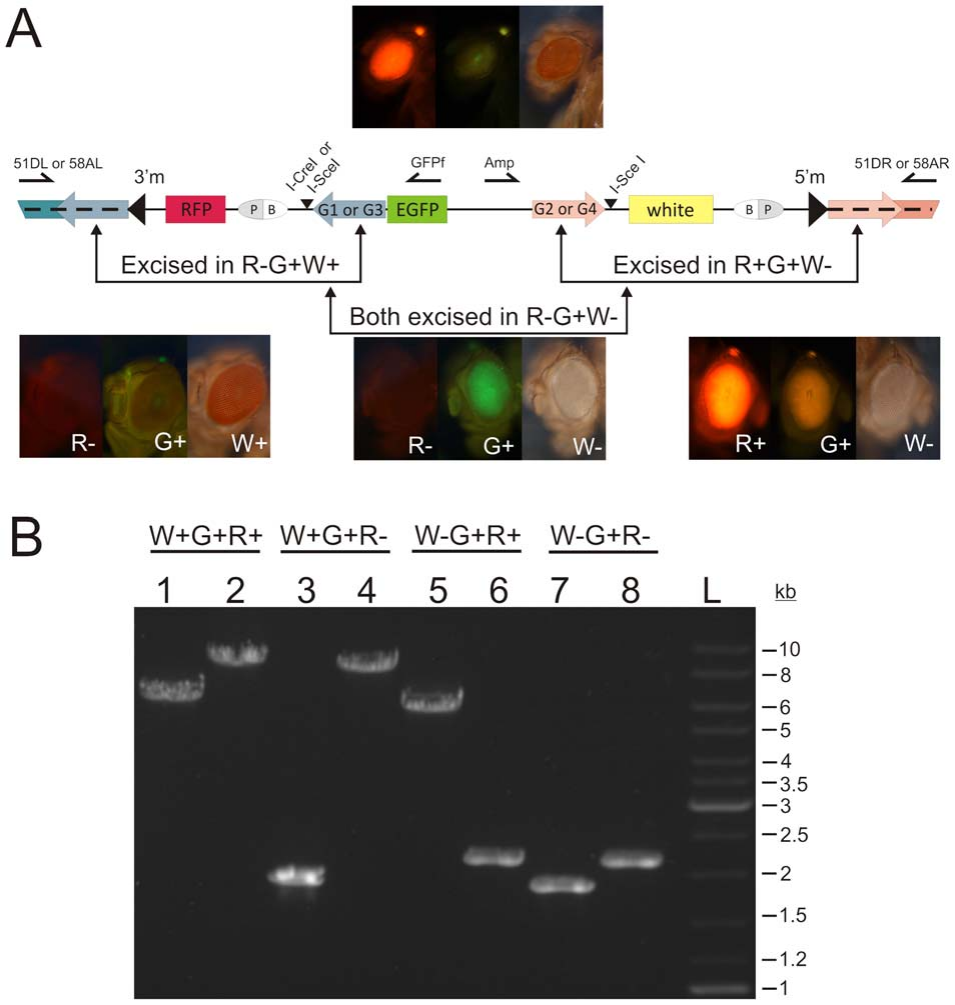
Phenotypes and PCR analysis of vector sequences in line TS51D and their derivatives. a. Combined TS51D, TS51D2xSce and TS58A2xSce schematic structure after integration in landing platform. Arrows above indicate primers. Image inserts show eye phenotypes of flies from TS51D2xSce line and their derivatives. b. Agarose gel with PCR products from indicated primers. Primer pairs 51DL/GFPf were used to analyze to analyze deletion of 3′ITR with RFP, Amp/51DR – deletion of 5′ITR with *white*. Primers pairs: lanes 1, 3, 5, 7 – 51DL/GFPf (prediction size of products 5630 or 1881 bp); lanes 2, 4, 8, 10 - Amp/51DR (7640 and 2167 bp). Size of PCR products for R-G+W+, R+G+W- и R-G+W- flies agree with the expected size for SSA-pathway repair (**Supplementary Table S3**).

We then collected 40 R+G+W- males from independent crosses and carried out individual crosses to females carrying a heat-inducible I-CreI source. I-CreI induction was performed only under light heat shock conditions, as heat shock can negatively affect fly survival [24]. The F2 resulting from this cross had 14.1% of flies that lost the RFP marker. In that case, DSB were introduced between G1 and RFP, and the 3′ITR of *mariner* was deleted along with RFP (**Fig. 1**). In these flies (R-G+W-), we could observe EGFP expression in the eyes, which was otherwise masked by RFP expression. For 30 R-G+W- flies from independent crosses, the repair products were verified by PCR analysis with the primer sets 51DL/GFPf and Amp/51DR, and eight flies were also confirmed by sequencing. In all cases, PCR products had an expected length of 1900 and 2200 bp (**Fig. 2**). Thus, the transgenic flies did not contain transposon *mariner* and marker genes, and the EGFP gene was integrated directly into the *D. melanogaster* genome between the *hibris* and *CG33467* genes. Sequencing revealed no remains of ITRs and therefore the possibility of EGFP remobilization would not exceed that of any other non-transposon fragments of the genome.

Despite the high efficiency of complete EGFP stabilization by sequential introduction of I-SceI and I-CreI sources, we hypothesized that simultaneous introduction of DSB will decrease the necessary number of fly generations, and would optimize our system. To this end, we introduced I-SceI and I-CreI sources in TS51D flies simultaneously. As expected, flies of the R-G+W- type appeared in the progeny, but only in small numbers (4/2302; (**Table 1**). This small population can be explained by the light heat shock conditions that are suitable for I-CreI, but are suboptimal for I-SceI induction.

To increase the effectiveness of full transgene stabilization achievable in one step, we created vector the TS51D2xSce, where the I-CreI-site between RFP and G1 was substituted with I-SceI-site. This vector was then integrated into the same landing site as TS51. After I-SceI source introduction, the TS51D2xSce flies were then exposed to strong, intermediate, or light heat shock after which 5.99%, 1.56%, and 0%, respectively, of flies were R-G+W- (**Table 1**). For 10 R-G+W- flies from independent crosses, we verified the repair products by PCR analysis and sequencing (**Fig 2**). The phenotypic classes R+G+W- and R-G+W+ were present in all three experiments. Together, these classes represented 35.36% under strong, 10.92% under intermediate and 4.4% under light heat shock.

To demonstrate the effectiveness of our method for different genome locations, another landing platform, *M{3xP3-RFPattP}ZH-58A* (2R: 17733123) was chosen. Thus, we created the vector TS58A2xSce, which is identical to TS51D2xSce in structure, but contains G3 (1000 bp) and G4 (442 bp) sequences instead of G1 and G2, respectively. G3 and G4 are homologous to the sequences flanking the landing platform. After strong heat shock induction of I-SceI in the transformants, 46.17% of F2 flies lacked one of the two markers (R+G+W- or R-G+W+) and 7.94% of F2 flies lost both markers(R-G+W-) (**Table 1**). DNA from 56 R-G+W- flies from 30 independent crosses was analyzed by PCR with primers 58AL/GFPf or Amp/58AR. The PCR product size and five independent sequences supported that the marker genes and *mariner* were eliminated by SSA (**Supplementary Fig. S1a**). Because EGFP was weak in this locus, whether EGFP was present and intact in these flies was also checked by PCR (data not shown). More than half of the progeny (54.12%) carried the stabilized transgene, and nearly one in ten flies had the EGFP gene indelibly integrated in the *D. melanogaster* genome between two *tRNA* genes.

## Discussion

The *phiC31* integrase system has been used successfully in human and mouse tissue culture cells and *in vivo* in mice [25], [26], [27]. This integrase system was also recently demonstrated to be functional in yellow fever mosquito *Aedes aegypti,* a disease-vector species [28] and the agricultural pest species Mediterranean fruit fly *Ceratitis capitata* [15]. According to our data, transgene integration occurs with high frequency (**Supplementary Table S2**), which corresponds with published data [19], [22], [23]. As reported previously, nonspecific integration is a very rare event [19], [23]. Even if such transformants were by chance to be chosen, they would be discarded following molecular analysis of stabilized lines or, even more likely, earlier in the transformant characterization process. The site-specific manner of DNA integration is an indisputable advantage of the *phiC31* integrase system that allows prediction of transgene expression, which is very useful for transgenic insect engineering. Also, a *phiC31*-mediated approach allows the stable integration of DNA fragments larger than 100 kb [29], which substantially exceeds transposon-mediated transformation capabilities and can be used in complex multi-gene construct integration. Together, these characteristics of the *phiC31* system ensure its broad implementation in transgenic insect engineering.

We chose two different landing platforms that have different expression of *white*: high in 51D and low in 58A [19]. As expected, integration of our vectors resulted in bright-orange eyes in 51D and pale-yellow with a mosaic phenotype eyes in 58A, suggesting a different transcriptional status of the surrounding chromatin. Regardless of the integration site, transgene stabilization frequency at optimal I-SceI expression was extremely high and reached 41.35% and 54.12% for TS51D2xSce and TS58A2xSce, respectively (**Table 1**).

The rare-cutting homing endonucleases I-SceI and I-CreI have extended recognition sites: 18 bp for I-SceI and 22 bp for I-CreI [16], [17], with I-SceI being widely used in DSB repair research in plants, animals and human cell lines [30], [31], [32], [33], [34]. I-SceI recognition sites are not found in the *D. melanogaster* genome, which facilitated its use in DSB introduction to unique sites of the genome. In contrast, a I-CreI recognition site in the *D. melanogaster* genome is located in the *28S rDNA* gene, leading to lethality when the endonuclease is highly expressed [24]. The use of rare-cutting endonucleases in other organisms can therefore introduce breaks into endogenous sequences and subsequently induce mutations. Our data on two-step stabilization using I-CreI suggests that the presence of recognition sites does not necessarily interfere with our method, although additional fitness tests might be necessary. An increased specificity could be achieved by using alternative endonucleases, such as artificial zinc-finger nucleases [35], [36]. Careful endonuclease selection is thus an important factor in determining the success of transgene stabilization. Modern full-genomic sequencing technologies will likely assist the search for suitable endonucleases and allow for screening of mutations in loci that could be affected by endonuclease action. Results from a recently launched interlaboratory project that aims to sequence over the next 5 years the genomes of 5,000 insects and related arthropod species important for agriculture, medicine and biotechnology [37] should be highly useful for such genomic screens. On the other hand, transposase-mediated remobilization is well-known to be a potentially mutagenic process. Before a transgene reaches its final place or is removed from the genome it can be ‘cut and pasted’ several times, which could leave deletions or duplications in its temporal locations. Such mutations are practically impossible to trace.

The success of SSA can depend on the duplication length and size of spacer between them [18], [31], [38]. We used duplicated sequences of different lengths: 439 bp (G4), 645 bp (G2), 998 bp (G1 and G3). Surprisingly, the highest yield of flies with the stabilized transgene was obtained for TS58A2xSce, when SSA caused the deletion of the shortest G4 sequence, a 4986 bp spacer (**Table 1**). Our data correspond with the fact that DSB between direct repeats larger than 147 bp will be repaired primarily by SSA [18], [31], [33], [39]. Among the flies without marker genes that we checked, we found none where genes were lost due to partial deletion and subsequent repair by the nonhomologous end joining (NHEJ) pathway.

We optimized our system by the simultaneous introduction of two DSB in the integrated vector. Moreover, when the I-CreI site was substituted with the second I-SceI, the effectiveness was increased 35-fold and reached almost 6% (8% for 58A locus). After introduction of two DSBs, a loss of essential sequences between them can be expected, so we tested for the presence of EGFP and whether the I-SceI site were intact in 87 flies having a W+(G+)R+ phenotype and found that all the flies carried EGFP. Most of the I-SceI sites in the W+(G+)R+ class were either not cut, or repaired in a precise manner. An insignificant number of flies without I-SceI sites were observed. Possibly, the sites in those flies were damaged during NHEJ repair (**Supplementary Fig. S1b**). We believe that the absence of EGFP deletion results from the introduction of homologous sequences into the vector that direct the repair primarily through the SSA pathway. Taken together, these data suggest a low possibility of the gene of interest being lost upon the simultaneous introduction of two DSB, and demonstrates the potential for stabilizing genes having no visible manifestation.

The transgenic construct *M{3xP3-RFPattP}* that we used as a landing platform is based on the mobile *mariner* element [19]. *Mariner*–based vectors can be used to produce various transgenic organisms, including different insect and vertebrate species [9]. However, after integration in *D. melanogaster* or *Ae. aegypti* genomes, transposons are known to demonstrate unexpected stability [40], [41]. Similar behaviors have also been observed for *piggyBac* and *Minos* in *Ae. aegypti* and *Anopheles stephensi* [42], [43]. This characteristic of the transposon makes transposase-mediated transgene stabilization very labor-intensive, which highlights the critical need to identify a universal vector that is capable of both insertion into a host genome and remobilization. Our stabilization approach is an alternative technology that only requires the vectors to have the capacity to integrate effectively.

The approach described here allows the generation of insects with transgenes that are integrated directly in the genome and do not contain unwanted DNA. Due to the method's high efficiency, insects with stabilized transgenes can be obtained literally in only a few test tubes. The use of *phiC31* integrase and I-SceI endonuclease, which effectively function in different organisms, along with the conservative SSA repair pathway, suggests that our method will be successful in stabilized transgene production in different insect species. This method will allow the generation of a wider range of transgenic insects for use in effective and environmentally-friendly pest management programs.

## Materials and Methods

### Plasmids

#### TS51D and TS51D2

Fragments G1, G2, G3 and G4 were PCR-amplified from genomic DNA with Kapa HiFi polymerase (Kapa Biosystems) and verified by sequencing. Oligonucleotides Sce1 (5′-GATCCATTACCCTGTTATCCCTAG-3′) and Sce2 (5′-GATCCTAGGGATAACAGGGTAATG-3′) were annealed and cloned into *BamHI*–cut pBluescript II SK (Stratagene), to obtain DNA fragment, containing I-SceI recognition site (pSK-Sce).The 4151 bp *EcoRI* fragment containing marker gene *white* was isolated from Caspew15 [44] and cloned into EcoRI site of pSK-Sce (pSK-SceW). The 645 bp G2 was cut with *SpeI* and *XbaI* and cloned into *SpeI*-cut pSK-SceW (pSKA) in such orientation that G2R was adjacent to I-SceI-site. Oligonucleotides Cre1(5′-CTAGACAAAACGTCGTGAGACAGTTTG-3′) and Cre2 (5′-GATCCAAACTGTCTCACGACGTTTTGT-3′) were annealed and cloned into the pSL1180 (Amersham Biosciences), digested with *XbaI* and *BamHI*, to obtain DNA fragment, containing I-CreI recognition site (pSLCre). The 998 bp G1 cleaved with *BamHI* and *SalI* and cloned into pSLCre, (pSLCreG1). Actin promoter was removed from pAcEGFP (kindly provided by O.Maksimenko) by digesting it with *BglII*, blunting and re-ligation of the plasmid (pEGFPΔp). 280bp 3xP3 promoter was amplified from p3xP3-DsRed1-orf (kindly provided by M.J. Fraser, University of Notre Dame) using primers 3xP3f and 3xP3r and cloned into pEGFPΔp as *EcoRI-NcoI* fragment (3xP3-EGFP). The plasmid was cut with *XbaI* and *NotI*, then blunted and ligated in order to remove these and *XhoI* restriction sites (3xP3-EGFPdeltaXX). EGFP under 3xP3 promoter was cloned as a 1308 bp *EcoRI-HpaI* fragment into pSLCreG1, digested with *SalI,* blunted and then digested with *EcoRI* (pSLB). pTAattB (kindly provided by F.Karsh) was *SpeI*-cut, Klenow-blunted and re-ligated (pTAattBΔSpeI). The attB recombination site was cloned as 374 bp *XhoI-SacI* fragment into pSL1180 (pSLattB) and then as 395 bp *XhoI-NruI* fragment into pSLB, yielding pSLBattB.DNA fragment, containing I-SceI recognition site was Klenow-blunted and cloned into the *NruI* site of pSLBattB to obtain an I-SceI site (pSLBattBSce).Finally, *white*-*ISceI*-G2 tandem was introduced into *XhoI* and *SpeI*-cut pSLBattB or pSLBattBSce, as 4843 bp *XhoI-SpeI* fragment from pSKA resulting in TS51D and TS51D2xSce.

*TS58A2xSce.* The 439 bp G2 fragment was cut with *SpeI* and *XbaI* and cloned into *SpeI*-cut pSK-SceW (pSKAG3) in such orientation that G2R was adjacent to I-SceI-site. G3 Fragment was *SalI* and *BamHI*-cut and and cloned into pSLCre, (pSLCreG3). EGFP under 3xP3 promoter was cloned as a 1308 bp blunted *EcoRI-HpaI* fragment from 3xP3-EGFPdeltaXX into blunted *SalI* site of pSLCreG1, (pSLBG3). *XhoI-NruI* fragment from pSLattB was cloned into *XhoI*, *NruI*-cut pSLBG3 yielding pSLBG3attB. Blunt DNA fragment, containing I-SceI recognition site was cloned into the *NruI* site of pSLBattB to obtain I-SceI site (pSLBG3attBSce). Finally, *white*-*ISceI*-G4 tandem was introduced into *XhoI* and *SpeI*-cut pSLBG3attBSce, as 4637 bp *XhoI-SpeI* fragment from pSKAG3 resulting in TS51D and TS51D2xSce.

### Drosophila strains and fluorescent marker detection

Strains containing I-CreI endonuclease were from Bloomington Drosophila Stock Center (stocks no. 6936 P{v[+t1.8] = hs-I-CreI.R}2A, v[1]; ry[506] and 6937 w[1118]; P{v[+t1.8] = hs-I-CreI.R}1A Sb[1]/TM6). Strain P{v+; hsp70-I-SceI}1A, carrying heat-inducible I-SceI nuclease transgene (hsp70-I-SceI) has been previously described by Rong and Golik (Rong & Golic 2003). Strains with landing platform and phiC31 integrase y[1] M{vas-int.Dm}ZH-2A w[*]; M{3xP3-RFP.attP}ZH-51D and y[1] M{vas-int.Dm}ZH-2A w[*]; M{3xP3-RFP.attP'}ZH-58A were kindly provided by F. Karsh [18]. Up to 100 pre-blastoderm embryos were microinjected with 500 ng/ul plasmid. Transformants with orange or pale-yellow eyes were selected. Fluorescent markers were detected *in vivo* under a Leica MZ16FA fluorescence stereomicroscope using TRITC filter set for RFP detection (exciter HQ545/30x; emission HQ610/75m; Chroma Technology) and GFP2 set for EGFP detection (excitation 480/40 nm; barrier 510 nm; Leica Microsystems). All Drosophila stocks were reared on a standard yeast medium at 25°C. Heat shock was carried out in three ways: strong – during the two days after eggs were laid for 2 hours at 37°C, intermediate – only on the following day for 1 hour at 37°C and light – only on the following day for 1 hour at 36°C. Details of genetic crosses are available from the authors upon request.

### Molecular analysis

Genomic DNA was isolated from individual flies of different phenotypes using standard phenol-chloroform method. PCR analysis of DSB-repair products was carried out with different primer sets (**Suplementary Table S3**) using Kapa2G Robust HotStart Polymerase (for products <2500 bp) or KAPA Long Range HotStart DNA Polymerase (for products >2500 bp) under manufacturer's instructions (Kapa Biosystems). The presence of EGFP and I-SceI sites in flies of W+(G+)R+ derivatives of TS58A2xSce was confirmed by PCR and restriction analysis as described in legend for **Supplementary Fig. S1b**.

## Supporting Information

Figure S1 PCR and restriction analysis of TS58A2xSce and its derivatives. a. Agarose gel with PCR products with primers, indicated on **Fig. 2**. Primer pairs 58AL/GFPf were used to analyze to analyze deletion of 3′ITR with RFP, Amp/58AR – deletion of 5′ITR with *white*. Primer pairs: lanes 1, 3, 5, 7 – 58AL/GFPf (prediction size of products 5740 or 1979 bp); lanes 2, 4, 8, 10 - Amp/58AR (7287 and 2051 bp). Size of PCR products for R-G+W+, R+G+W- и R-G+W- flies agree with the expected size for SSA-pathway repair. b. Detection of EGFP and I-SceI site in R+(G+)W+ flies collected after induction of I-SceI in TS58A2xSce. One sample agarose gel with 8 probes is shown, while 87 probes were analyzed. “-” mark lanes with PCR product amplified with primers attP/GFPf, surrounding the I-SceI recognition site between RFP and G3. 2400 bp PCR-product indicates presence of EGFP in the analyzed flies. “+“ mark lanes with PCR-product after digestion with I-SceI. Two restriction products of 1900 and 500 bp are detectible. PCR product in sample 3 is undigested due to mutation of I-SceI site in consequence of NHEJ-repair (lanes 5, 6).
(EPS)

Table S1 Statistical analysis of vectors stabilization data.
(DOC)

Table S2 Effectiveness of *phiC31*-mediated transformation of embryos.
(DOC)

Table S3 Predicted PCR products from TS51D, TS51D2xSce, TS58A2xSce inserted into landing platform and theirs derivatives.
(DOC)

Table S4 Sequence of primers used in molecular analysis and cloning.
(DOC)
