# Supplementary material for: A New Powerful Method for Site-Specific Transgene Stabilization Based on Chromosomal Double-Strand Break Repair
Source: PLoS One. 2011 Oct 17;6(10):e26422. doi: 10.1371/journal.pone.0026422 (PMC3195726; doi:10.1371/journal.pone.0026422)
Supplement: Table S1 — Statistical analysis of vectors stabilization data. (DOC) [file pone.0026422.s002.doc]

Table S1. Statistical analysis of vectors stabilization data.

|  | **TS51D** | | | | **TS51D2xSce** | | | **TS58A2xSce** |
| --- | --- | --- | --- | --- | --- | --- | --- | --- |
|  | **I-SceI** | | **I-CreI** | **I-SceI + I-CreI** | **I-SceI** | | | **I-SceI** |
|  | ***strong*** | ***light*** | ***light*** | ***light*** | ***strong*** | ***intermediate*** | ***light*** | ***strong*** |
| **Tests ^a^** | 43 | 15 | 40 | 40 | 22 | 22 | 25 | 30 |
| **Flies in test ^b^** | 88.90 ± 9.12 | 71.93 ± 12.47 | 91.20 ± 4.36 | 57.55 ± 7.63 | 53.09 ± 8.68 | 55.32 ± 7.06 | 57.83 ± 5.42 | 127.13 ± 16.23 |
| **Phenotype %** ± CI, P=0.95 | | | | | | | | |
| **W+(G+)R+** | 61.12 ± 5.03 | 94.22 ± 2.73 | - | 72.29 ± 4.91 | 54.68 ± 8.63 | 87.74 ± 7.36 | 95.72 ± 3.55 | 45.17 ± 4.42 |
| **W-(G+)R+** | 38.88 ± 5.03 | 5.78 ± 2.73 | 85.8 ± 3.97 | 1.21 ± 0.61 | 12.79 ± 4.47 | 4.64 ± 2.79 | 0.55 ± 3.73 | 29.62 ± 5.19 |
| **W+G+R-** | - | - | - | 26.37 ± 4.85 | 25.41 ± 7.01 | 6.34 ± 4.45 | 3.73 ± 3.33 | 17.52 ± 3.95 |
| **W-G+R-** | - | - | 14.2 ± 3.97 | 0.13 ± 0.19 | 7.12 ± 3.30 | 1.27 ± 1.75 | 0 | 7.7 ± 2.61 |
| **stabilization ^c^** | 38.88 ± 5.03 | 5.78 ± 2.73 | 100 ^d^ | 27.71 ± 4.91 | 45.32 ± 8.63 | 12.26 ± 7.36 | 4.28 ± 3.55 | 54.83 ± 4.42 |

**^a^**  Number of independent crosses.

^b^ average number of progeny in a cross ± CI (P=0.95).

^c^  summary stabilization after deletion of at least one ITR (sum of **W-(G+)R+, W+G+R- and W-G+R-)**

^d^ DSB induction by I-CreI endonuclease was carried out in flies with already stabilized transgene (without 3’ITR and *white*).
