# Supplementary material for: A New Powerful Method for Site-Specific Transgene Stabilization Based on Chromosomal Double-Strand Break Repair
Source: PLoS One. 2011 Oct 17;6(10):e26422. doi: 10.1371/journal.pone.0026422 (PMC3195726; doi:10.1371/journal.pone.0026422)
Supplement: Table S2 — Effectiveness of phiC31-mediated transformation of embryos. (DOC) [file pone.0026422.s003.doc]

Table S2. Effectiveness of phiC31-mediated transformation of embryos.

| Vector | Landing platform | Number of  injected embryos / fertile flies | Number of individual crosses yeilding transformants | ^a^Frequency of transgenesis (% ) |
| --- | --- | --- | --- | --- |
| TS51D | *ZH-51D* | 100 / 82 | 17 | 20.7 |
| TS51D2xSce | *ZH-51D* | 100 / 84 | 20 | 23.8 |
| TS58A2xSce | *ZH-58A* | 75 / 65 | 24 | 36.9 |

^a^We define the frequency of transgenesis as the fraction of fertile crosses that gave at least one *white+* offspring as described by Bischof et al [1].

1. Bischof J, Maeda RK, Hediger M, Karch F, Basler K (2007) An optimized transgenesis system for Drosophila using germ-line-specific phiC31 integrases. *Proc Natl Acad Sci USA* 104: 3312-3317.
