# Supplementary material for: A New Powerful Method for Site-Specific Transgene Stabilization Based on Chromosomal Double-Strand Break Repair
Source: PLoS One. 2011 Oct 17;6(10):e26422. doi: 10.1371/journal.pone.0026422 (PMC3195726; doi:10.1371/journal.pone.0026422)
Supplement: Table S3 — Predicted PCR products from TS51D, TS51D2xSce, TS58A2xSce inserted into landing platform and theirs derivatives. (DOC) [file pone.0026422.s004.doc]

Table S3. Predicted PCR products from TS51D, TS51D2xSce, TS58A2xSce inserted into landing platform and theirs derivatives.

| Primer pairs | Vector | Phenotype classes | | | |
| --- | --- | --- | --- | --- | --- |
|  |  | R+G+W+ | R-G+W+ | R+G+W- | R-G+W- |
| 51DL/GFPf | TS51D | 5630 | 1881 | 5630 | 1881 |
| Amp/51DR | TS51D | 7640 | 7640 | 2187 | 2187 |
| 51DL/GFPf | TS51D2xSce | 5650 | 1881 | 5650 | 1881 |
| Amp/51DR | TS51D2xSce | 7640 | 7640 | 2187 | 2187 |
| 58AL/GFPf | TS58A2xSce | 5740 | 1979 | 5740 | 1979 |
| Amp/58AR | TS58A2xSce | 7287 | 7287 | 2051 | 2051 |
