# Supplementary material for: A New Powerful Method for Site-Specific Transgene Stabilization Based on Chromosomal Double-Strand Break Repair
Source: PLoS One. 2011 Oct 17;6(10):e26422. doi: 10.1371/journal.pone.0026422 (PMC3195726; doi:10.1371/journal.pone.0026422)
Supplement: Table S4 — Sequence of primers used in molecular analysis and cloning. (DOC) [file pone.0026422.s005.doc]

Table S4. Sequence of primers used in molecular analysis and cloning.

| Primer | Sequence |
| --- | --- |
| G1F | TGGATCCATTCTGAAATGAATTAAGAAGCAGT |
| G1R | GTAGTCGACAAAGGCTATAATCCAAGAATTTA |
| G2F | TACTAGTAAAAGGGTGGGATGTGACG |
| G2R | ATTCTAGAGCCATGGGGCTATGGTTCA |
| G3F | TGGATCCAGGCATACTTTTAGGGGTC |
| G3R | GTAGTCGACATTATATAGCTAAACCGTTATT |
| G4F | TACTAGTAGATATTAACAGCAAAATGCAAT |
| G4R | GGCTCTAGACCGCGTTACG |
| 51DL | GCAATCAAAGAGTCGACGACTTTTG |
| 51DR | AATAAATGGGCGGGTAAGCA |
| 58L | AGGCGAGCATGCAAATTAG |
| 58R | CAGCGTCTGGTTTTGCGATA |
| Amp | CTGTCATGCCATCCGTAAGATGCTTTTC |
| GFPf | ACACCCTGGTGAACCGCATCGA |
| 3xP3f | ATgAATTCgAgCTCgCCCggggAT |
| 3xP3r | ATACCATgggTggCgACCggTggAT |
| attP | gtactgacggacacaccgaa |
